# Recurrent Glioblastoma: What Is the Route?

**DOI:** 10.3390/cancers15072028

**Published:** 2023-03-29

**Authors:** Alberto Bosio, Giuseppe Lombardi

**Affiliations:** Department of Oncology, Oncology 1, Veneto Institute of Oncology IOV-IRCCS, via Gattamelata 64, 35128 Padua, Italy; alberto.bosio@iov.veneto.it

Glioblastoma (GBM) is the most frequent and aggressive malignant primary central nervous system tumor in adults. The standard of care for newly diagnosed GBM patients is represented by the Stupp protocol, which consists of maximal safe resection, when feasible, and concomitant chemoradiation followed by adjuvant temozolomide. At relapse, which virtually occurs for all GBM patients, there is no general consensus about the optimal second-line treatment.

Re-surgery may be evaluated in selected cases, and it has shown low morbidity and minimal impact to cognitive functions in the short-term period after surgery [1].

An additional, although technically demanding, intraoperative therapeutic option is represented by interstitial photodynamic therapy using 5-aminolevulinic acid (5-ALA) as the photosensitizer. Siller et al. retrospectively demonstrated that when performed by expert hands, this technique is associated with low morbidity and the possibility of long survival [2].

Conventional systemic therapy with nitrosoureas and temozolomide rechallenge has limited efficacy, thus leading to an increasing trend toward precision medicine and targeted therapy for GBM.

NTRK and FGFR inhibitors and the combination of BRAF and MEK inhibitors are some of the most promising treatment options that have been investigated [3]. Moreover, regorafenib has been confirmed in a real-life randomized phase 2 study REGOMA as being a promising therapeutic option for recurrent GMB [4]. Regorafenib is also being studied in an ongoing Italian phase 1 study (REGOMA-2) in association with temozolomide and radiotherapy as a first-line therapy in newly diagnosed glioblastoma patients. On the other hand, Khurshed et al. demonstrated no clinical response of metformin and chloroquine in combination in patients with IDH-1 mutated gliomas in a phase Ib study [5].

Immunotherapy with checkpoint inhibitors, such as pembrolizumab, failed to improve overall survival in patients with complete or partial loss of mismatch protein expression [6].

GBM, indeed, is characterized by a high proportion of immunosuppressive innate immune cells, including GBM-associated microglia/macrophages (GAMs), and a limited number of effector cells that, when present, are dysfunctional. Recent studies have suggested that a GBM-immune microenvironment may be reprogrammed to enhance responsiveness to immunotherapy [7].

Network-based studies, which ultimately can lead to the identification of key genes in the development, progression and drug resistance stages, are crucial for improving personalized medicine and developing new therapeutical strategies [8].

Another interesting approach toward developing new treatments for GBM patients relies on microRNAs (miRNAs). miRNAs are endogenous small non-coding RNA which regulate gene expression by targeting mRNA molecules. Several studies have reported that miRNAs are involved in GBM tumorigenesis.

Indeed, some miRNAs are gene silencers of anti-apoptotic genes and inhibit the growth and survival of GBM. Therefore, the expression or function of miRNAs may serve as potential therapeutic strategies for GBM treatment. In addition, since many MiRNAs support cell growth in GBM, they might also represent a powerful diagnostic tool for the stratification of poor prognosis GBM [9].

Although new therapeutic options for GBM are still in their infancy, several studies are in progress. To achieve these goals, preclinal studies are needed. Campolo et al. demonstrated in an in vivo xenograft model and in temozolomide-treated patients that the inhibition of transforming growth factor-beta-activated kinase-1 (TAK1), an essential component in mitogen-activated protein kinase (MAPK) pathways, enhances the sensitivity of GBM cells to temozolomide and chemotherapy in general [10]. TP5, a small peptide which specifically inhibits tumor-related cyclin-dependent kinase 5 (CDK5)/p25 activity, showed in in vivo models a synergistic effect with either temozolomide or radiation due to an accumulation of DNA damage [11]. Ranjan et al. highlighted that MTUS1/ATIP1 modulates tumor progression by reducing the proliferation and motility of high-grade glioma cells and that a high level of ATIP1 might interfere with radiation therapy, since it causes double-strand break DNA repair [12].

Not only the treatment of relapsed GBM but also the diagnosis of pseudoprogression post-chemoradiation remain challenging tasks. Lohman et al. in their study proved that O-(2-[^18^F]fluoroethyl)-L-tyrosine (FET) positron emission tomography (PET) radiomics can be a powerful tool to discriminate between progression and pseudoprogression [13].

As it is now well determined that GBM stem cells play a relevant role in tumor resistance and recurrence, several authors have reported their findings on stem-cell-focused studies. Vieira de Castro et al. demonstrated that intracellular autofluorescence can identify GMB cells that display stem cell features, thus representing an inexpensive way to target this tumor, with clinical and research implications [14]. Another factor that impacts therapy resistance and, consequently, the poor prognosis of patients affected by GBM is tumor heterogeneity. Liesche-Starnecker and colleagues found new varieties of the GBM subtype via immunochemistry and cluster analysis. In particular, they showed that these subtypes cannot only be found in different GBMs, but that they coexist in the same tumor and vary in primary and relapsed tumors [15]. Interestingly, Zao and colleagues also evaluated the GBM environment by establishing a GBM mouse model. Particularly, the authors found that monocyte-derived macrophages (MDMs) are the prevalent myeloid population at recurrence, versus microglia being the most represented in primary GBM, and that this characteristic was not affected by pharmacological or surgical therapy [16]. Additionally, for the first time, La Rocca et al. performed a proteomic analysis of cavitating ultrasound aspirator (CUSA) fluid. The authors reported that a portion of protein profiles is shared by the tumor core and in the tumor periphery, as defined by the presence or absence of 5-aminolevulinic acid fluorescence. The share of protein expression in 5-aminolevulinic acid fluorescence tumor zones could account for the aggressiveness and infiltrative nature of GBM [17].

Carbon ion irradiation (CIR) may be a novel therapeutic option for recurrent high-grade gliomas. Knoll et al. performed a whole blood transcriptome analysis via liquid biopsy for monitoring the longitudinal molecular changes that occur under this type of therapy [18]. Another irradiation option, photodynamic therapy (PDT) using talaporfin sodium (NPe6) (NPe6-PDT), was recently approved in clinical practice. In their work, Kobaiashi and colleagues demonstrated that under this therapy, a more malignant phenotype of GBM is induced by the activation of the ERK1/1 pathway, thus representing a possible promising candidate therapeutic target for GBM [19].
